# Functional definition of BirA suggests a biotin utilization pathway in the zoonotic pathogen *Streptococcus suis*

**DOI:** 10.1038/srep26479

**Published:** 2016-05-24

**Authors:** Huiyan Ye, Mingzhu Cai, Huimin Zhang, Zhencui Li, Ronghui Wen, Youjun Feng

**Affiliations:** 1 College of Life Science and Technology, Guangxi University, Nanning City, Guangxi 530004, China; 2 Department of Medical Microbiology and Parasitology, Zhejiang University School of Medicine, Hangzhou, Zhejiang 310058, China

## Abstract

Biotin protein ligase is universal in three domains of life. The paradigm version of BPL is the *Escherichia coli* BirA that is also a repressor for the biotin biosynthesis pathway. *Streptococcus suis*, a leading bacterial agent for swine diseases, seems to be an increasingly-important opportunistic human pathogen. Unlike the scenario in *E. coli, S. suis* lacks the *de novo* biotin biosynthesis pathway. In contrast, it retains a *bioY*, a biotin transporter-encoding gene, indicating an alternative survival strategy for *S. suis* to scavenge biotin from its inhabiting niche. Here we report functional definition of *S. suis birA* homologue. The *in vivo* functions of the *birA* paralogue with only 23.6% identity to the counterpart of *E. coli*, was judged by its ability to complement the conditional lethal mutants of *E. coli birA*. The recombinant BirA protein of *S. suis* was overexpressed in *E. coli*, purified to homogeneity and verified with MS. Both cellulose TLC and MALDI-TOFF-MS assays demonstrated that the *S. suis* BirA protein catalyzed the biotinylation reaction of its acceptor biotin carboxyl carrier protein. EMSA assays confirmed binding of the *bioY* gene to the *S. suis* BirA. The data defined the first example of the bifunctional BirA ligase/repressor in *Streptococcus*.

Biotin (vitamin H) is one of two known sulfur-containing fatty acid derivatives (biotin^1^,^2^ and lipoic acid^3^), and acts as an enzyme cofactor universal in three domains of the life. Although the biotin-requiring enzymes are rare proteins (in that mammals have only four such proteins whereas *Escherichia coli* has only a single biotinylated protein)^2^,^4^, they play critical roles in certain important reactions (like carboxylation, decarboxylation and trans-carboxylation) implicated into fatty acid synthesis, gluconeogenesis and amino acid degradation in both prokaryotes and eukaryotes^5^,^6^. Right now, it is aware that most microorganisms (bacteria and fungi) and plants possess the ability to synthesize biotin, whereas mammals and birds cannot^4^. The earlier steps of biotin synthesis are involved in a modified type II fatty acid synthesis pathway in *E. coli*^7^,^8^, whereas the latter of biotin synthesis route refers to a highly-conserved four-step reactions catalyzed by BioF, BioA, BioD and BioB, respectively^6^,^9^. In light that biotin is an energetically-expansive molecule in that generally 15–20 ATP equivalents are estimated to be consumed via its paths of *de novo* synthesis for each biotin^4^, it seems reasonable that bacteria have developed diversified mechanisms to tightly monitor the level of biotin production *in vivo*^1^,^4^,^6^,^10^,^11^. In addition to the paradigm *E. coli* BirA regulatory system that also retains the activity of biotin-protein ligase^12^,^13^,^14^,^15^, at least two more regulatory machineries have been reported^1^,^4^,^10^,^11^,^16^. Among them, one is the two-protein system of BirA coupled with BioR, the GntR-family transcription factor^1^,^4^,^11^, and the other denotes the two-protein system of the BirA linked to BioQ, a TetR family of transcription factor^10^,^16^.

In fact, bacteria have evolved two different mechanisms to obtain the biotin cofactor for the metabolic requirement, one of which is *de novo* synthesis route^2^,^11^, the other is a system of BioY transporter-mediated uptake^5^. Unlike the human pathogen *Brucella*, a member of α-proteobacteria that encodes the above two systems for the availability of biotin^11^, it seems likely that the species of *Streptococcus*/*Lactococcus* family only have the BioY-based scavenging route and compensates the lack of the *de novo* biotin synthesis pathway^17^. Among microorganisms, the paradigm enzyme with the biotin requirement refers to biotin carboxyl carrier protein (abbreviated as BCCP, *i.e*., the AccB subunit of acetyl-CoA carboxylase (ACC)) that catalyzes the first committed reaction for type II fatty acid synthesis pathway^18^. Biotin protein ligase (BPL) is widespread in three domains of the life in that it transfers/attaches the biotin cofactor to the specific domain of the relevant subunits of key enzymes from the certain central metabolisms^19^,^20^. Most of bacteria including *E. coli*^14^,^18^ and *Bacillus*^21^ only encode a single BPL to account for such kind of physiological requirement, while the pathogen *Fracisella novicida* developed an additional BPL to gain the competitive advantage in the infected host environment^2^. In general, the BPL members are categorized into the following two groups (Group I and Group II) that can be easily distinguished by the presence of N-terminal DNA-binding domain that allow the BirA protein to bind the cognate genes (*e.g*., *bio* operon), and thereafter inhibit expression of biotin metabolism^19^,^21^. Unlike the paradigm Group II BPL proteins, the *E. coli birA* gene product retaining the DNA-binding activity, the Group I BPL that lacks the N-terminal helix-turn-helix domain solely function as an enzyme responsible for protein biotinylation^6^,^9^. In particular, the regulatory role of the Group II BPL depends on the participation of the physiological ligand/effector (biotinoyl-5′-AMP), the product of the first ligase half reaction for biotin utilization/protein biotinylation^12^.

*Streptococcus suis* (*S. suis*) is a leading agent of bacterial diseases including meningitis, arthritis and septicemia) in swine industry worldwide^22^,^23^, and also appears to be an opportunistic zoonotic pathogen responsible for human *S. suis* infections such as meningitis and even streptococcal toxic shock-like syndrome (STSS)^24^. Given the difference of bacterial capsule, 35 kinds of serotypes (1–34, 1/2) have been attributed to *S. suis*^25^. Among them, the serotype 2 of *S. suis* is frequently isolated from diseased piglets and highly-related to the strong virulence^22^,^23^,^25^. *S. suis* 2 (SS2) is a previously-neglected, but newly-emerging human pathogen, claiming a series of occupational/ opportunistic infections. Since the first discovery of human SS2 meningitis was recoded in Denmark, in 1968^25^, SS2 has spread to nearly 30 countries (and/or regions) and caused no less than 1,600 human cases^26^. In particular, two big-scale outbreaks of fatal human SS2 infections had ever emerged in China (one in Jiangsu Province, 1998, and the other in Sichuan Province, 2005), posing a great concern to public health^24^,^27^,^28^. In 2007, we also reported three sporadic cases of human SS2 meningitis in China (two cases in Shenzhen City, and one case in Chongqing City)^29^, implying the co-existence of outbreaks and sporadic cases in China^24^,^26^,^28^,^29^. It is unusual that the epidemic strain of Chinese virulent SS2 harbors a pathogenicity island (PAI) referred to 89 K^30^,^31^. Subsequent functional exploration suggested that this 89 K PAI behaves as a transposon-like genetic element^32^ and encodes type IV secretion system^33^ and SezAT toxin-antitoxin system^34^. Our epidemiological investigation argued that the 89 K PAI might be undergoing unknown selective pressure in that some variants losing 89 K PAI are emerging^35^. The remodeling of bacterial surface structure significantly was found to attenuate full virulence of the epidemic SS2 strain^36^. Very recently, we observed that regulation of the D-galactosamine (GalN)/N-acetyl-D-galactosamine (GalNAc) catabolism pathway is linked to its infectivity^37^. It seems likely that the regulatory network of bacterial metabolism is complicated into SS2 virulence^26^,^38^. Given the fact that biotin metabolism and utilization is associated with *Francisella* pathogenesis^2^,^39^,^40^, it is much interest to define the biotin utilization pathway in the human pathogen *S. suis* 2.

In this paper, the epidemic SS2 strain in China, *S. suis* 05ZYH33 with the known genome sequence was subjected to the context analyses of the bacterial biotin metabolism and its possible regulation. Unlike the scenarios seen with the paradigm organism *E. coli*, the possible biotin machinery in the *S. suis* we detected comprises a single BioY (SSU05_0509) transporter regulated by the BirA bifunctional protein (05SSU_1625) and the biotin-requiring substrate protein AccB (SSU05_1801). By employing integrative approaches that ranged from comparative genomics, bioinformatics, biochemistry/biophysics, metabolomics, to bacterial genetics, we attempted to present a full picture of biotin utilization pathway in the zoonotic pathogen *S. suis*.

## Results and Discussion

### *S. suis* BirA Protein is a Group II BPL Member

**Figure 1:**
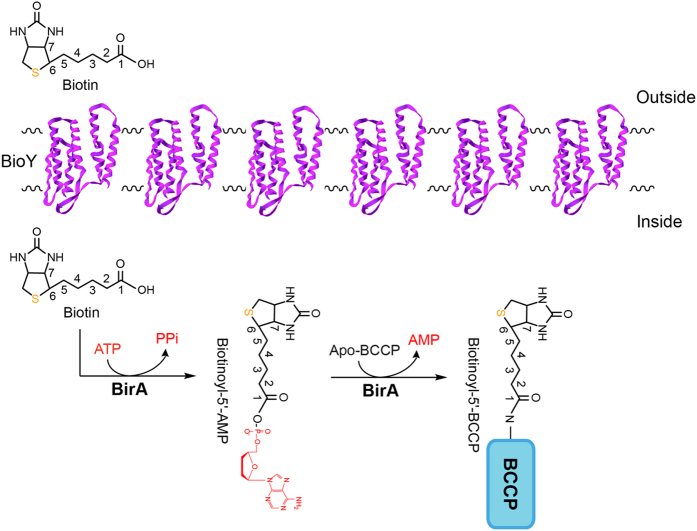
Current model for biotin transport and utilization in *Streptococcus suis.* The biotin transporter BioY was illustrated with modeled ribbon structure (in purple) and integrated into the scheme of bacterial membrane. The biotin transported from environment was activated into biotinoyl-5′-AMP and then transferred to BCCP acceptor protein giving the biotinoyl-5′-BCCP. Sulphur was labeled in orange, and AMP (and/or ATP/PPi) was highlighted in red. The biotin acceptor protein BCCP was indicated with a blue rectangle.

**Figure 2:**
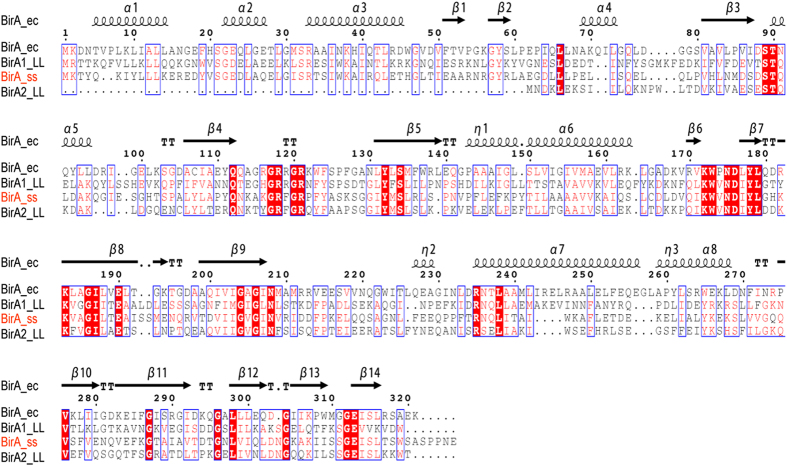
Sequence comparison of *Streptococcus suis* BirA orthologue with the prototypical *E. coli* version. The multiple sequence alignment of BirA protein was performed using the program of Clustal Omega, an updated version of ClustalW2 (http://www.ebi.ac.uk/Tools/msa/clustalo), and the final output was given with the program ESPript 3.0 (http://espript.ibcp.fr/ESPript/cgi-bin/ESPript.cgi). Identical residues are white letters with red background, similar residues are red letters in white background, the varied residues are in grey letters, and gaps are denoted with dots. The protein secondary structure was shown in cartoon (on top), α: α-helix; β: β-sheet; T: Turn; η: coil.

**Figure 3:**
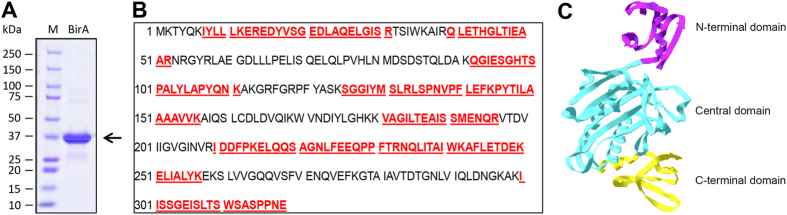
Characterization of *S. suis* BirA protein. (**A**) SDS-PAGE profile of the purified recombinant BirA protein from *Streptococcus suis* Gradient SDS-PAGE (4–20%) was applied to separate the protein. (**B**) MS-based determination of the recombinant protein of *S. suis* BirA The peptide fragments that matched the native form of *S. suis* BirA were given with bold and underlined letters (in red). Totally, 54% coverage was detected. (**C**) Modeled ribbon structure of *S. suis* BirA protein *S. suis* BirA protein included three domains: the N-terminal DNA-binding domain (in purple), the central domain (in blue), and the C-terminal domain with enzymatic activity (in yellow).

It seems likely that *S. suis* is biotin auxotrophic in that it is deficient in biotin synthesis, and depends on the mechanism of BioY-BirA to scavenge biotin from the inhabiting niche and/or infected host environment (Fig. 1). System biology by Rodionov *et al*.^41^ revealed that bi-functional BPL enzymes (Group II) exemplified with the *E. coli* BirA, are universal in both Eubacteria and Archaea, implying the group II form might be the ancestor of the BPL. Unlike the group I without DNA-binding domain BPL (*e.g.*, BirA orthologues of *Agrobacterium*^4^ and *Brucella*^11^), the multiple sequence alignment analyses suggested that *S. suis* BirA is generally similar to the paradigm *E*. coli *birA* product (Fig. 2). However the situation seemed unusual in the close-relative of *S. suis, Lactococcus lactis* in that this probiotic bacterium contained two versions of BPL, one of which refers to BirA1__LL_ (Group II BPL) and the other is BirA2__LL_ lacking the DNA-interacting motif (Group I BPL) (Fig. 2). To address the BPL biochemistry of the *S. suis* BirA, we applied protein engineering to produce the recombinant protein. As anticipated, we harvested the BirA_ss protein with the mass of around 37 kDa (Fig. 3A). Also, the purity was judged with SDS-PAGE (Fig. 3A). To further assure the identity, the polypeptide fragments digested from the recombinant BirA protein were subjected to the analyses of MALDI-TOFF. The MS result suggested that the recombinant protein matched well the native form of the *S. suis* BirA in that it exhibited the coverage of 54% (Fig. 3B). Structural modelling assigned the *S. suis* BirA as a typical version of the group II BPL with the perfect architecture (Fig. 3C). It comprised the following three functional motifs: N-terminal DNA-binding domain, Central domain and C-terminal domain (Fig. 3C). Obviously, the above data defined the *S. suis* BirA as a member of Group II BPL.

### Activity of Biotin Protein Ligase of *S. suis* BirA

**Figure 4:**
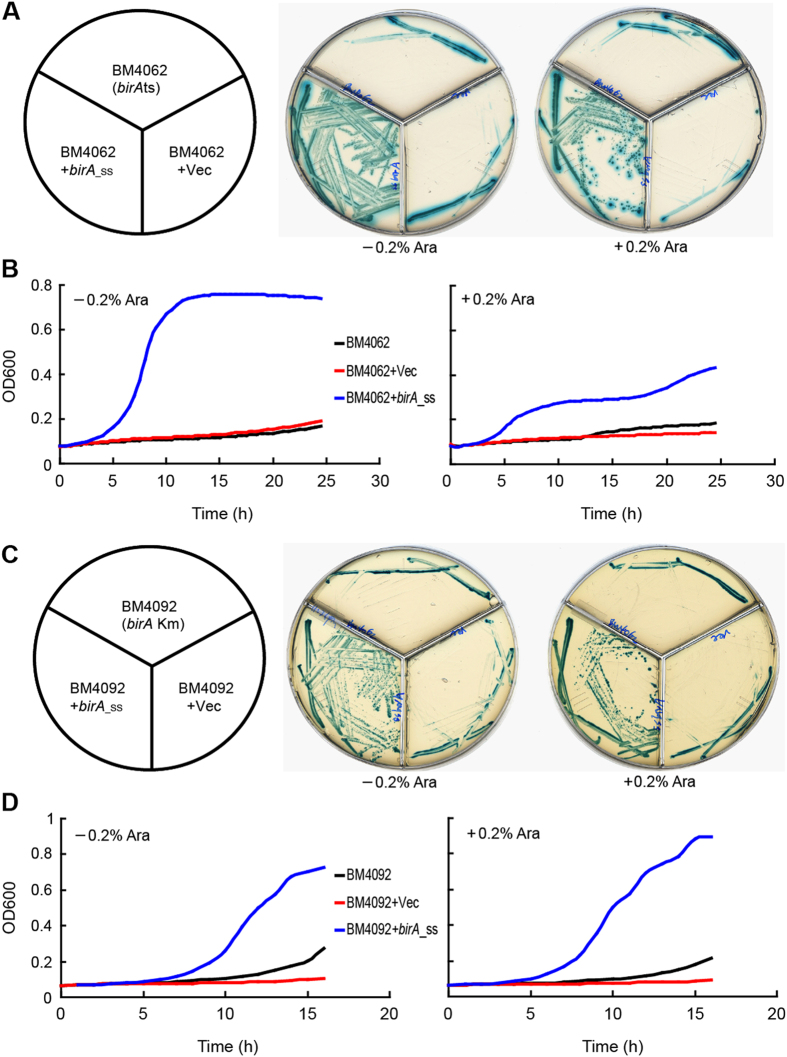
Functional complementation of *E. coli birA* mutants with the putative biotin protein ligase-encoding gene *birA.* (**A**) Growth of *E. coli* BM4062 *birA*ts mutant carrying plasmid-borne *S. suis birA* gene on the M9 agar plates (**B**) Growth curves of *E. coli* BM4062 *birA*ts mutant and its derivatives *E. coli* strains were maintained at 42 °C (the non-permitted temperature of BM4062) on the defined media M9 with/without 0.2% arabinose for around 36 hours. M9 agar plates were supplemented with 0.5 mM X-gal plus 100 nM biotin. (**C**) Growth of *E. coli* BM4092 *birA*1 Km mutant carrying plasmid-borne *S. suis birA* on the M9 agar plates. (**D**) Growth curves of *E. coli* BM4092 *birA*1 Km mutant and its derivatives. *E. coli* strains were maintained at 30 °C on the defined media M9 with/without 0.2% arabinose for around 36 hours. M9 agar plates were supplemented with 0.5 mM X-gal plus 25 nM biotin. The bacterial growth was measured by optical density at 600 nm, which is automatically recorded using a *BioScreen C* instrument. Each growth curve assay was carried out in triplicate and the average was used in this plot^50^. Designations: Vec, vector (c); ts: temperature-sensitive; Ara, Arabinose.

We employed the *in vitro* and *in vivo* approaches to address the BPL activity of *S. suis* BirA. The two *E. coli birA* mutants used for functional complementation included the temperature-sensitive mutant of *birA*ts (BM4062) and the *birA*1 Km mutant (BM4092). As expected, the BM4062 Strain with/without the empty vector pBAD24 cannot grow on the M9 agar plates under the non-permissive temperature of 42 °C (Fig. 4A). In contrast, the arabinose-induced expression (and even basal expression) of the plasmid-borne *birA*_ss supported the growth of the *birA*ts mutant BM4062 at 42 °C (Fig. 4A). The measurement of bacterial growth curves for BM4062 strains in liquid media also reproduced the similar results to those obtained from the agar plates (Fig. 4B). The presence of pBAD24 plasmid-borne *birA*_ss allowed the *birA*1 Km mutant of *E. coli*, BM4092 to grow on the minimal media supplemented with low level of biotin (25 nM) (Fig. 4C), which agreed well with the scenario seen in the growth curves (Fig. 4D). Obviously, it confirmed that *S. suis* BirA has a role of being the BPL ligase in the alternative model, *E. coli*.

**Figure 5:**
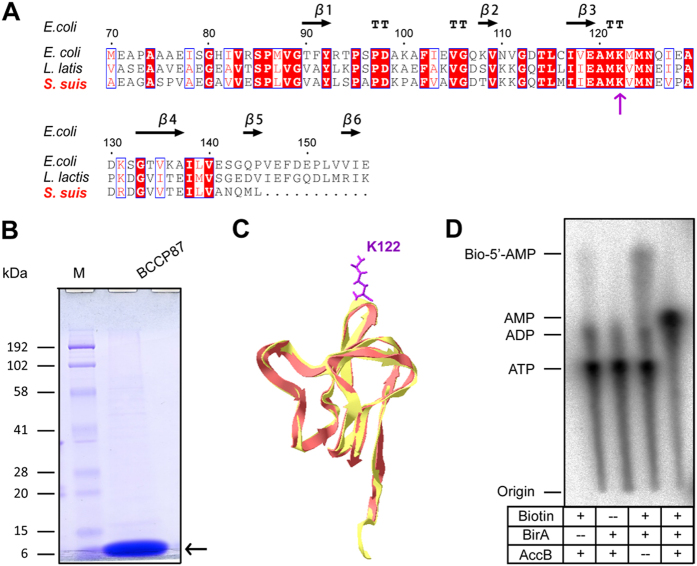
Evidence that *S. suis* BirA biotinylates the AccB substrate protein. (**A**) Sequence analyses for the biotinylation domain of the BCCP acceptor protein (also referred to AccB). (**B**) SDS-PAGE profile of the biotin substrate domain (BCCP87 or AccB87). (**C**) Structural modeling of the biotinylated domain of the *S. suis* AccB protein The biotinylation site of AccB is lysine at the position 122 (K122). (**D**) TLC assays for the BPL enzymatic activity of *S. suis* BirA. The abbreviations: M, protein marker (Biorad); kDa, kilo-dalton. Plus (+) and Minus (−) denotes presence and absence of BirA protein, Biotin or AccB87 substrate protein.

To further prove the BPL function of *S. suis* BirA, we established the assays of enzymatic reaction *in vitro*. In this system of BirA-catalyzed reaction, the substrate used is biotinylated domain (designated as AccB87 or BCCP87) of the AccB protein (Fig. 5A,B), which carries a conserved biotinylation site of lysine at the position 122 (K122) (Fig. 5A,C). The method of thin layer chromatography (TLC) was applied to assay conversion of α-^32^P-labeled ATP and biotin to biotinoyl-AMP (Fig. 5D). In principle, it represents direct evidence for the first ligase partial reaction (Fig. 5D), and upon an addition of the acceptor protein AccB87 provides an indirect proof of the second ligase partial reaction (*i.e.*, transferring of biotin from biotinoyl-5′-AMP to the AccB87 acceptor protein) (Fig. 5D). As expected, the *S. suis* BirA protein was shown to convert biotin and [α-^32^P]-ATP to the canonical biotinoyl-5′-AMP intermediate (Fig. 5D) and transferred the biotin moiety to the AccB-87 acceptor protein (Fig. 5D).

**Figure 6:**
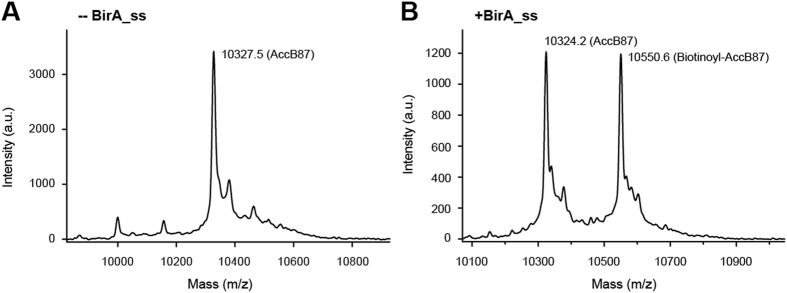
MS-based verification for *S. suis* BirA-catalyzed biotinylation of the AccB87 substrate protein. (**A**) MALDI-TOF determination of the molecular weight for the un-biotinylated AccB87 polypeptide. (**B**) MALDI-TOF identification for the biotinylation of the AccB87 substrate by *S. suis* BirA. The calculated mass for AccB87 is 10324.2~10327.5, and the expected mass for biotinoyl-AccB87 by *S. suis* BirA (Panel B) is 10550.6. Designations: minus denotes no addition of BirA enzyme, plus denotes addition of the BirA protein.

Subsequently, we utilized the matrix-assisted laser desorption/ionization (MALDI) to measure the level of BirA ligase-catalyzed biotinylation of AccB-87 as we recently described^4^. The MS results illustrated that the calculated mass for AccB87 is 10324.2~10327.5 (Fig. 6A), and the expected mass for biotinoyl-AccB87 is 10550.6 (Fig. 6B). Collectively, the integrative data demonstrated that *S. suis* BirA acts as a functional member of the BPL family.

### Binding of *S. suis* BirA to the cognate *bioY* gene

**Figure 7:**
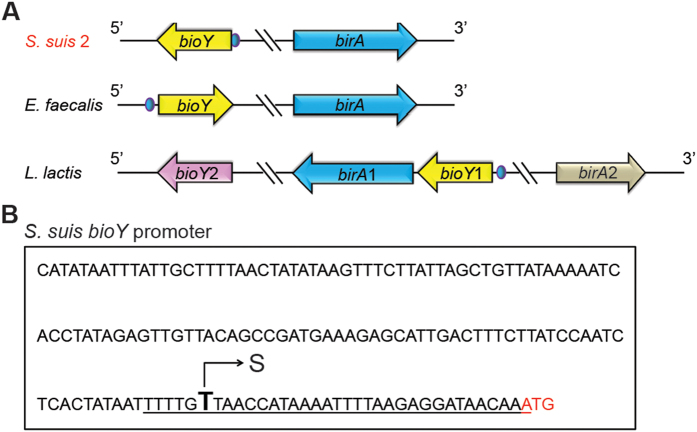
Genetic organization of *birA* (and *bioY*) and *S. suis bioY* promoter. (**A**) Genetic organization of *birA* (and *bioY*). The locus of *birA* (or referred to *birA*1) and *bioY* (referred to *bioY*1) is highlighted in blue and yellow, respectively. The blue spot denotes the predicted BirA-binding site. The locus of *birA*2 and *bioY*2 is indicated with an arrow in grey and purple. (**B**) *S. suis bioY* promoter. “S” denotes the putative transcriptional start site, and “ATG” in red is translation initiation site. The predicted BirA-binding site is underlined.

It is unusual that *S. suis* does not have the ability of *de novo* biotin synthesis in that this zoonotic pathogen lacks the *bio* operon found in *E. coli*^6^ and other organisms like *Agrobacterium*^4^ and *Paracoccus*^1^. However, it seemed likely that the inability of *S. suis* to make biotin is compensated with the BioY-mediated biotin uptake/scavenging pathway (Figs 1 and 7A). Also, the *bioY* lous is present in other two closely-relatives of the human pathogen (*Enterococcus faecalis* and *Lactococcus lactis*) (Fig. 7A). In particular, the *L. lactis* encoded two versions of *bioY* genes as well as two orthologues of BirA (Fig. 7A), implying the complexity of biotin metabolism in the certain species of low-GC contents, gram-positive bacteria.

**Figure 8:**
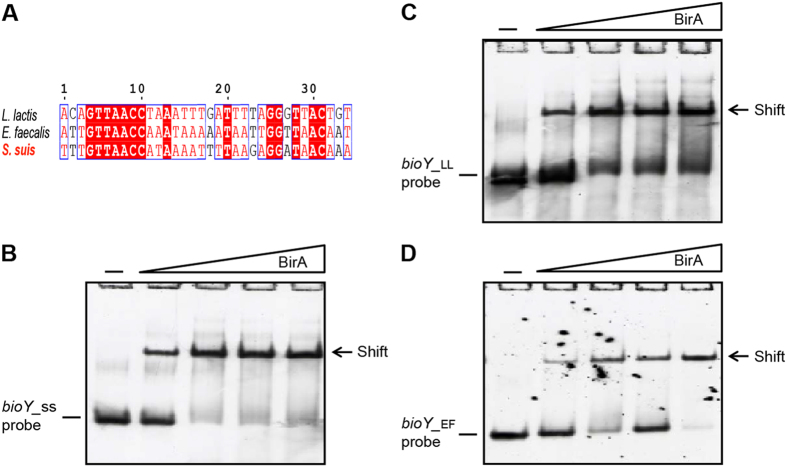
Interplay between BioY and BirA. (**A**) Multiple sequence alignment of the BirA-binding sites. (**B**) BirA protein of *S. suis* binds to its own *bioY* promoter. (**C**) Binding of *S. suis* BirA to the *L. lactis bioY* promoter. (**D**) Interaction of *S. suis* BirA with the *E. faecalitis bioY* promoter. Using 7% native PAGE, gel-shift assays were conducted, and a representative photograph is given. In each assay, levels of BirA are denoted with a triangle on right hand (0.1, 0.5, 2 and 5 pmol), whereas all the DIG-labeled probes (*bioY*_SS, *bioY*_LL, and *bioY*_EF) are added to 0.2 pmol. Minus sign denotes no addition of BirA protein. Designations: SS, *Streptococcus suis*; LL, *Lactococcus lactis*; EF, *Enterococcus faecalitis.*

The transcription start site of the *S. suis bioY* gene is estimated to be “T” that is 29 bp ahead of the translation initiation site “ATG”. Bioinformatics analyses suggested that a putative BirA-binding site (TTT TGT TAA CCA TAA AAT TTT AAG AGG ATA ACA A) covering the transcription start site is present in the *S. suis bioY* promoter region (Fig. 7B). Given the above observations, we proposed that the *bioY* might be negatively regulated by the *S. suis* BirA. While this hypothesis required experimental evidence. We tested the ability of BirA to bind *bioY* promoter using a 54 bp probe containing the predicted site using the electrophoretic mobility shift (gel shift) assays (Fig. 8A) as we recently performed^2^ with minor modifications. Gel shift assays showed that *S. suis* BirA efficiently bound the *bioY* probe in a dose-dependent manner (Fig. 8B) in that nearly 100% of *bioY* probe was transferred into the DNA-protein complex in the presence of 0.5 pmol BirA (Fig. 8B). In light of the appreciable conservation of the BirA sites in the *bioY* promoter from the related organisms (Fig. 8A), we also examined possible crosstalk of the BirA to bioY of various origins. In fact, the *S. suis* BirA was found to exhibit comparable binding to the *bioY* probes of *L. lactis* (Fig. 8C) and *E. faecalitis* (Fig. 8D). It demonstrated that physical interaction is present between the BirA bifunctional protein and the biotin transporter-encoding gene *bioY*.

### Physiological Implications for Biotin Utilization Pathway

**Figure 9:**
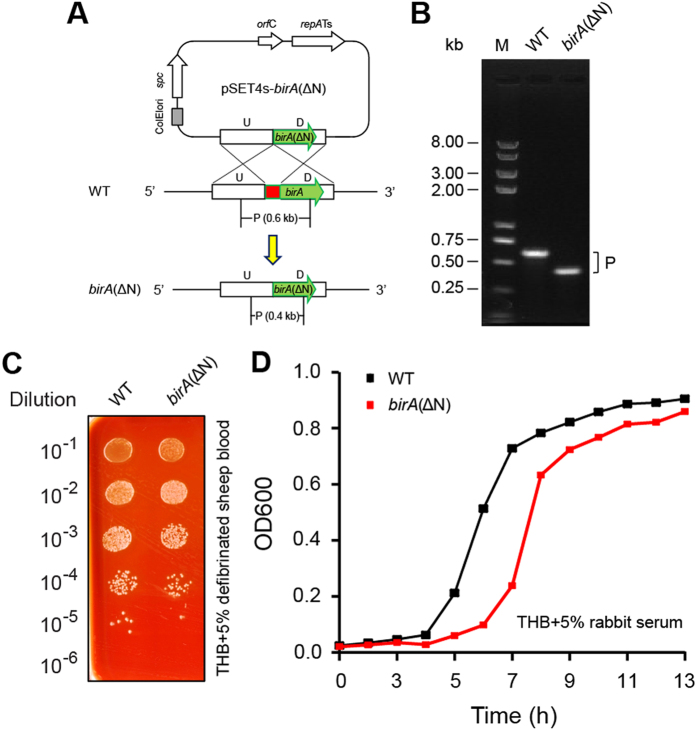
The *birA*(ΔN) mutant of *S. suis.* (**A**) Schematic for the in-frame deletion of the N-terminal DNA-binding domain from the *S. suis birA*. (**B**) PCR assays for the *birA*(ΔN) mutant of *S. suis.* The phenotype of the *birA*(ΔN) mutant of *S. suis* when growing on the THB plate (**C**) and in the liquid media (**D**).

It is reasonable that *S. suis* deficient in biotin synthesis evolved the mechanism of BioY-BirA to utilize the biotin scavenged from the inhabiting niche and/or infected host environment (Fig. 1). In the epidemic strain of *S. suis* serotype 2, 05YH33, three genes with the involvement of biotin metabolism denote *bioY* (SSU05_0509), *birA* (05SSU_1625), and *accB* (05SSU1801), respectively. Following the biotinylation by BirA, the AccB was converted from its apo-form into holo-form, and participated into the initiation of fatty acid biosynthesis. Given the fact that BirA binds the *bioY* promoter with the help of biotinoyl-5′-AMP (the intermediate of biotin biotinylation), the regulatory function of the BirA protein is supposed to guarantee that the wasteful production of the BioY transporter is avoided/minimized upon the biotin uptake from the outside environment. Probably, it is a physiological advantage for certain species of *Streptococcus*/*Lactococcus* in the context of lipid metabolism. To test above anticipation, we constructed the *birA*(ΔN) mutant of *S. suis* of which the DNA-binding domain was in-frame deleted (Fig. 9A,B). The removal of N-terminal DNA-binding motif affect bacterial growth on THB media, but this growth defection can be rescued by supplementation of the 5% defibrinated blood (or blood sera) (Fig. 9C,D). However, the expression of the plasmid-borne *bioY* promoter-driven lacZ is not altered significantly in the *birA*(ΔN) mutant in relative to the wild type (not shown). It might suggest a possibility that the interplay between BirA and *bioY* represent a developing and/or degenerating system for *S. suis*.

## Conclusions

Our data shown here defined a working model for the route of biotin uptake/utilization in the zoonotic pathogen *S. suis* 2 (Fig. 1). Unlike the scenarios observed in both *Brucella*^11^ and *Paracoccus*^1^ in that the *bioY* gene interacts with the BioR regulator, our finding represents a first example for the interplay between the *bioY* and BirA in the *Streptococcus*/*Lactococcus*. Of note, no reaction is present between the *bioY* gene and BioR in the plant pathogen *Agrobacterium*^4^, a close relative of the human pathogen *Brucella*^11^. It suggested the complexity and diversity of bacterial biotin metabolism and regulation. Given the fact that the biotin synthetic genes *bioJ*^39^ and *bpl*^2^ are involved in bacterial virulence of the intracellular pathogen *Francisella novicida*, it is of much interest to probe the possible role of biotin metabolism in *Streptococcus* pathogenesis. While the fact that both *bioY* and *birA* are essential for bacterial viability of *Streptococcus suis* argued the technical feasibility in the genetic removal of the two biotin-related genes. As we knew, biotin and lipoic acid both are sulfur-containing vitamins required for the three domains of the life. Similarly, the scavenging of lipoic acids by LplA was also required for the intracellular growth/survival and virulence^42^,^43^. Thereby we screened the genome sequence of *S. suis* 05ZYH33 for the presence of the *lplA* gene that encodes lipoate-protein ligase, giving the perfect hit (SSU05_1836). We are planning to examine its relevance to bacterial infectivity. Right now, it seemed true that both biotin and lipoic acid are nutritional virulence factors for certain species of bacterial pathogens. Given the fact that *S. suis* 2 is an emerging/reemerging infectious agent threatening public health^26^, our finding might be helpful to better understanding biology and even infection of this zoonotic pathogen.

## Methods

### Bacterial strains and growth conditions

Bacterial strains used here included *E. coli* and *Streptococcus suis* (Table 1), and all the *E. coli* strains are derived from the wild-type K-12 (Table 1). The two media (Luria Bertani (LB) and rich broth (RB)) were utilized for *E. coli*, whereas the Todd Hewitt Broth (THB) medium was used for *S. suis*^37^. Antibiotics were supplemented as follows (in mg/liter): sodium ampicillin, 100; kanamycin sulfate, 50; and Spectinomycin, 100.

### Plasmids and genetic manipulations

The *birA* gene (SSU05_1625) was amplified by PCR with genomic DNA of *S. suis* 05ZYH33 as the template, and cloned into the expression vector pET28(a), giving the recombinant plasmid pET28-*birA*_ss (Table 1). To prepare the BirA protein, the expression plasmid pET28-*birA*_ss was transformed into the strain BL21(DE3), giving the strain FYJ280 (Table 1)^44^.

Also, the *birA*_ss gene was cloned into the arabinose-inducible expression vector pBAD24^4^, giving the plasmid pBAD24-*birA*_ss. To evaluate the *in vivo* activity of BirA, two *birA* mutants of *E. coli* were applied, which referred to the *birA* km mutant strain BM4092, and the temperature-sensitive mutant BM4062, respectively (Table 1). Given the fact the *birA* is a bifunctional gene and is required for bacterial viability, it is reasonable to delete the partial function of *birA* at 5′-end. Therefore we employed an approach of homologous recombination to remove the N-terminal DNA-binding domain from the *birA* gene of *S. suis* 05ZYH33, giving the mutant *birA*(ΔN) (Table 1). In this case, a thermos-sensitive suicide vector pSET4s^45^ was applied. The promoter of *S. suis bioY* was fused to the promoter-less *lacZ* gene, creating the plasmid-borne P*bioY*-*lacZ* fusion (Table 1). To examine role of *birA in vivo*, the P*bioY*-*lacZ* fusion was separately introduced into the wild-type strain and the *birA*(ΔN) mutant of *S. suis*. All the acquired plasmids were verified by the PCR assay and direct DNA sequencing.

### Expression and purification of BirA protein

The *E. coli* carrying the 28-*birA*ss was used for preparation of the recombinant protein of *S. suis* BirA. The bacterial cultures were induced with 0.5 mM isopropyl β-D-1-thiogalactopyranoside (IPTG) at 30 °C for 3 h. The clarified bacterial supernatant was loaded onto a nickel affinity column (Qiagen). The 6x His-tagged protein of interest was eluted in elution buffer containing 150 mM imidazole, and the purity was judged with SDS-PAGE.

### Liquid chromatography quadrupole time-of-flight mass spectrometry

A Waters Q-Tof API-US Quad-ToF mass spectrometer was applied to determine the identity of *S. suis* BirA (BirA_ss) protein^1^,^46^. The purified protein band was cut from the gel and digested with Trypsin (G-Biosciences St. Louis, MO), giving a pool of overlapping peptides loaded on a Waters Atlantis C-18 column (0.03 mm particle, 0.075 mm × 150 mm). The acquired data were subjected to the ms/ms analyses.

### *In vitro* Bio-5′-AMP synthesis and thin-layer chromatography

The *in vitro* assay was established to determine the protein biotinylation activity of BirA ligase as we described previously^47^ with some modifications. The system of enzymatic reactions included 50 mM Tris-HCl (pH 8), 5 mM tris-(2-carboxyethyl) phosphine, 5 mM MgCl_2_, 20 μM biotin, 5 μM ATP plus 16.5 nM [α-^32^P]ATP, 100 mM KCl and 2 μM ligase protein. The reaction mixtures were maintained at 37 °C for 30 min. To figure out the role of the BirA ligase, two tubes of reaction were kept in parallel, only one of which was supplemented with AccB-87 (50 μM). Subsequently, 1 μl of each reaction mixture was spotted on a cellulose thin-layer chromatography plate of microcrystalline cellulose and the plates were developed in isobutyric acid-NH_4_OH-water (66:1:33 by volume)^48^. The thin-layer chromatograms were dried overnight, exposed to a phosphor-imaging plate and visualized using a Fujifilm FLA-3000 Phosphor Imager.

### MALDI-based determination for the biotinylation activity of BirA

The reaction of BirA-catalyzed biotinylation comprised the following components (100 μM AccB-87, 3 μM ligase, 100 μM biotin, 1 mM ATP, 10 mM MgCl_2_, 100 mM KCl, 5 mM tris-(2-carboxyethyl) phosphine in 50 mM Tris-HCl (pH 8.0)). The reactions were kept at 37 °C for 16 h then dialyzed against 25 mM ammonium acetate, lyophilized to dryness. Subsequently, the biotinylated form of the AccB87 from the above samples was assayed using the approache of matrix-assisted laser desorption/ionization (MALDI)^4^.

### Electrophoretic mobility shift assays

Gel shift experiments were conducted to test interaction of BirA protein with the *bioY* promoters of different origins^44^,^46^,^49^. Three sets of DNA probes ((*bioY*_SS, *bioY*_LL, and *bioY*_EF) were prepared by annealing two complementary oligonucleotides (Table 2). In the EMSA trials, the digoxigenin-labeled DNA probes (~0.2 pmol) were incubated with the purified BirA_ss protein in the binding buffer (Roche). When necessary, the biotinyl-5′-AMP ligand was supplemented. The DNA/protein mixtures were separated with the native 7% PAGE and transferred onto nylon membrane by the direct contact gel transfer, giving the chemical-luminescence signals captured via the exposure of the membrane to ECL films (Amersham).

### β-Galactosidase assays

Overnight cultures of *S. suis* carrying the *lacZ* fusion grown in THB medium were subjected to measure direct measurement of β-galactosidase activity^44^. When necessary, the blood sera were added to augment bacterial growth of the mutant *S. suis*. The bacterial lysates were prepared using French pressure. The data were recorded in triplicate more than three independent assays.

### Bioinformatics analyses

The orthologues of BirA protein were from *E. coli, Lactococcus lactis*, and *S. suis* 05ZYH33, respectively. The BirA-binding sites were collected from RegPrecise database (http://regprecise.lbl.gov/RegPrecise/regulon.jsp?regulon_id=53141). Using the program of ClustalW2 (http://www.ebi.ac.uk/Tools/clustalw2/index.html), the multiple alignment of protein (and/or DNA) were carried out, and the final outputs were given with the program ESPript 2.2 (http://espript.ibcp.fr/ESPript/cgi-bin/ESPript.cgi). The transcription start site was predicted using the server of Neutral Network Promoter Prediction (http://www.fruitfly.org/seq_tools/promoter.html). Structural modelling was proceeded with CPHmodels 3.2 Server (http://www.cbs.dtu.dk/services/CPHmodels).

## Additional Information

**How to cite this article**: Ye, H. *et al*. Functional definition of BirA suggests a biotin utilization pathway in the zoonotic pathogen *Streptococcus suis. Sci. Rep.* **6**, 26479; doi: 10.1038/srep26479 (2016).

## Figures and Tables

**Table 1:** Bacteria and plasmids used in this study.

Strains or plasmids	Relevant characteristics	Origins
**Strains**		
Topo10	Cloning host of *E. coli*	Invitrogen
BL21 (DE3)	Expression host of *E. coli*	Lab stock
BM4092	*birA* km mutant of *E. coli*	4
BM4062	*birA*ts temperature-sensitive mutant of *E. coli*	4
FYJ277	Topo 10 carrying pET28a-*birA_*ss	This work
FYJ278	Topo 10 carrying pBAD24-*birA_*ss	This work
FYJ279	BL21 carrying pET28b-LLbccp87	This work
FYJ280	BL21 carrying pET28a-*birA_*ss	This work
05ZYH33	An epidemic strain of Chinese virulent SS2	Lab stock
*birA*(ΔN)	The *birA* mutant of 05ZYH33 lacking the N-terminal DNA binding domain	This work
**Plasmids**		
pSET4s	A thermosensitive suicide vector, Spc^R^	45
pET28a(+)	T7 promoter-driven expression vector for production of recombinant protein in *E. coli*	Novagen
pBAD24	An arabinose-inducible expression vector	51
pET28a-*birA*_ss	pET28a carrying the *S. suis birA* gene	This work
pBAD24-*birA*_ss	A derivative of pBAD24 encoding the *S. suis birA* gene	This work
pSET4s-UD	A derivative of pSET4s for in-frame deletion of N-terminal domain of the *S. suis birA*	This work

**Table 2:** Primers used in this study.

Primers	Sequences
*SSbirA*-F1	5′-CG *GGATCC* ATG AAA ACC TAT CAG AAA ATA T-3'
*SSbirA*-R1	5′-CCG *CTCGAG* TTA TTC GTT TGG TGG AGA AGC-3'
*SSbirA*-F2	5′-AACC *GAATTC* ATG AAA ACC TAT CAG AAA ATA T-3'
*SSbirA*-R2	5′-CCG *GTCGAC* TTA TTC GTT TGG TGG AGA AGC-3'
SS*birA-bioY*-F	5′-CAC TAT AAT T**TT TGT TAA CCA TAA AAT TTT AAG AGG ATA ACA AA**T GAA AAC AAC-3'
SS*birA-bioY*-R	5′-GTT GTT TTC A**TT TGT TAT CCT CTT AAA ATT TTA TGG TTA ACA AA**A ATT ATA GTG-3'
*LLbirA*-*bioY*-F	5′-CAA ATA ATA AAA TTA **ACA GTT AAC CTA AAT TTG ATT TTA GGG TTA CTG T**TT GAT ATG-3'
*LLbirA*-*bioY*-R	5′-CAT ATC AA**A CAG TAA CCC TAA AAT CAA ATT TAG GTT AAC TGT** TAA TTT TAT TAT TTG-3'
*EFbirA*-*bioY*-F	5′-CCG CTA AAC T**AT TGT TAA CCA AAT AAA AAT AAT TGG TTA ACA AT**A GAA AGT GAG-3′
*EFbirA*-*bioY*-R	5′-CTC ACT TTC T**AT TGT TAA CCA ATT ATT TTT ATT TGG TTA ACA AT**A GTT TAG CGG-3′
*birA*-U-F	5′-CCC AAGCTT CCA TCT CTT TCA GCC AAG GC-3′
*birA*-U-R	5′-CGG GTA GGA GCA TAC CTT CAT TAT AGC AAA AA-3′
*birA*-D-F	5′-TGA AGG TAT GCT CCT ACC CGA ATT AAT CTC T-3′
*birA*-D-R	5′-G GAATTC TTT ATG AAT GCA TCG ATT TTG AC-3′
P-F	5′-CAC CAC CTT TAG CGG TCT TA-3′
P-R	5′-TGG TAT TTA TAT GTC GCT TCG T-3′

The underlined letters in italic are restriction sites, and the bold letters denote the BirA-binding sites.
